# A Distributed Parallel Genetic Algorithm of Placement Strategy for Virtual Machines Deployment on Cloud Platform

**DOI:** 10.1155/2014/259139

**Published:** 2014-07-03

**Authors:** Yu-Shuang Dong, Gao-Chao Xu, Xiao-Dong Fu

**Affiliations:** ^1^College of Computer Science and Technology, Jilin University, Changchun 130012, China; ^2^Key Laboratory of Symbolic Computation and Knowledge Engineering of Ministry of Education, Jilin University, Changchun 130012, China

## Abstract

The cloud platform provides various services to users. More and more cloud centers provide infrastructure as the main way of operating. To improve the utilization rate of the cloud center and to decrease the operating cost, the cloud center provides services according to requirements of users by sharding the resources with virtualization. Considering both QoS for users and cost saving for cloud computing providers, we try to maximize performance and minimize energy cost as well. In this paper, we propose a distributed parallel genetic algorithm (DPGA) of placement strategy for virtual machines deployment on cloud platform. It executes the genetic algorithm parallelly and distributedly on several selected physical hosts in the first stage. Then it continues to execute the genetic algorithm of the second stage with solutions obtained from the first stage as the initial population. The solution calculated by the genetic algorithm of the second stage is the optimal one of the proposed approach. The experimental results show that the proposed placement strategy of VM deployment can ensure QoS for users and it is more effective and more energy efficient than other placement strategies on the cloud platform.

## 1. Introduction

Cloud computing is at the forefront of information technology. The internal system of cloud computing can be seen as a collection of a set of services [1], including infrastructure layer (IaaS), platform layer (PaaS), and application layer (SaaS). With the development of cloud computing, more and more cloud centers provide IaaS as the main way of operating. In order to improve the utilization rate of cloud center and to decrease the operating costs, virtualization technology has been applied to the cloud computing [2–4]. It provides services as required to users by sharding the resources with virtualization. But the distribution of virtual machines (VMs) will become sparser on cloud center with creating and closing the VMs. The placement problem of VMs has attracted more and more attention and became a research hotspot in cloud computing area quickly. It can be regarded as packing problem and has been proved as a NP-completeness problem [5].

Most of early researches were focused on increasing resources utilization rate in considering the system performance. With the increase of cloud center scale, energy saving has attracted significant attention in both industry and academia area. In order to reduce operating costs by saving energy, the concept of green cloud is proposed. Most researches are focused on VMs consolidation with living migration technology to reduce energy costs. If we take the energy costs into consideration as a parameter in the VMs deployment process, it can effectively reduce live migration frequency for decreasing the energy costs in maintenance of cloud center.

Genetic algorithm has been appreciated by academic circles as a solution of the VMs placement problem because of its speediness and adaptability advantages. Furthermore, parallel genetic algorithm can be used to solve the relatively complex problems. Even so, the genetic algorithm probably terminates before it gets a good enough solution in the case that there are a large number of servers in cloud platform and users need to deploy a certain number of VMs. The traditional parallel genetic algorithm is executed on single physical host, but Amdahl's law [6] showed that the performance of parallel program executed on single physical host is not much better than serial program. The researches [7, 8] showed that we can get a better performance of parallel program by enlarging the scale of problem. Therefore, we propose a new distributed parallel genetic algorithm (DPGA) of placement strategy which is executed on several physical hosts for the large-scale VMs deployment problem. This algorithm can get a better and more accurate solution by increasing the iterative times. Comparing with the deployment process, the time cost of deployment strategy is relatively less. Therefore, we did not take the time cost in consideration in DPGA. We define the initial population of the DPGA as initial total population and the initial population of algorithm executing on each selected host as initial subpopulation. We assign the performance per watt as fitness value. In order to ensure the coverage of the solution space, we choose initial subpopulation from solution space dispersedly and averagely. It executes the first stage genetic algorithm on several selected physical hosts to choose initial subpopulation and get several solutions. Then it collects the solutions calculated by the first stage of DPGA and puts them into the second stage as initial population. Finally, we get a relatively satisfied solution from the second stage of DPGA.

## 2. Relevant Work

The proposed question refers to finding the target hosts to place the VMs. In this paper, relevant knowledge of DVFS will be used in the standard for evaluating the solution in considering of minimizing energy costs and ensuring performance as well. This subject has not been widely studied in the field related to placement strategy for VMs deployment. However, many researches are focused on the placement of applications and services in the cloud environment [9–15], and many researchers have been working on data placement in the cloud center [16–19].

There are also some researches focused on the similar problems. von Laszewski et al. have presented a scheduling algorithm to allocate virtual machines in a DVFS-enabled cluster [20]. The proposed algorithm was focused on scheduling virtual machines in a compute cluster to reduce power consumption via the technique of DVFS (dynamic voltage and frequency scaling). It dynamically adjusts the CPU frequencies and voltages of the compute nodes in a cluster without degrading the virtual machine performance beyond unacceptable levels. Recent studies have revealed that the network elements consume 10–20% of the total power in the data center. VMPlanner [21] optimized both virtual machine placement and traffic flow routing so as to turn off as many unneeded network elements as possible for network power reduction in the virtualization-based data centers. It took the advantage of the flexibility provided by dynamic VM migration and programmable flow-based routing to optimize network power consumption while satisfying network traffic demands. Ge et al. have presented distributed performance-directed DVS (dynamic voltage scaling) scheduling strategies for use in scalable power-aware HPC (high-performance computing) clusters [22]. It uses DVS technology in high-performance microprocessors to reduce power consumption during parallel application runs in the case that peak CPU performance is not necessary due to load imbalance, communication delays, and so forth. VMPACS [23] is a multiobjective ant colony system algorithm for the virtual machine placement problem. The purpose of VMPACS is to efficiently obtain a nondominated Pareto set that simultaneously minimizes total resource wastage and power consumption. MILP [24] proposed a holistic approach for a large-scale cloud system where the cloud services are provisioned by several data centers interconnected over the backbone network. It is a mixed integer linear programming formulation that aims at virtualizing the backbone topology and placing the VMs in inter- and intradata centers with the objective of jointly optimized network delay and energy saving. OVMP [25] is an optimal virtual machine placement algorithm to provision the resources offered by multiple cloud providers. It is based on an IaaS model which leverages virtualization technologies that can minimize the total cost of resource in each plan for hosting virtual machines in a multiple cloud provider environment under future demand and price uncertainty. The tradeoff between the advance reservation of resources and the allocation of on-demand resources is adjusted to be optimal. It makes a decision based on the optimal solution of stochastic integer programming (SIP) to rent resources from cloud providers. Jing Tai Piao proposed a network-aware virtual machine placement and migration approach for data intensive applications in cloud computing environments to minimize the data transfer time consumption [26]. It places the VMs on physical machines with consideration of the network conditions between the physical machines and the data storage. It also considers the scenario in which instable network condition changed the data access behaviors and deteriorated the application performance. It migrates the VM to other physical machines in order to deal with this scenario.

## 3. Distributed Parallel Genetic Algorithm of VMs Placement

There are *w* physical hosts in the cloud platform and users need *n* VMs with (*h*_0_, *h*_1_, *h*_2_,…, *h*_*n*−1_) Hz CPU and (*m*_0_, *m*_1_, *m*_2_,…, *m*_*n*−1_) M RAM. We assume that the physical hosts in cloud center are DVFS [27] enabled and the cloud center can satisfy requirements of users; namely, *w* is big enough for *n*. We assume that *w* ≫ *n* and the physical hosts are in the same network environment. Solution space is recorded as follows: *P* = (*P*_0_, *P*_1_, *P*_2_,…, *P*_*w*−1_). We need to find *n* physical hosts to satisfy the requirements of users to place the VMs. The solution vector is as follows: *S* = (*S*_0_, *S*_1_, *S*_2_,…, *S*_*n*−1_). Remaining available CPU resource of physical host *P*_*i*_ is as follows: AF_*i*_ = (1 − *U*_*i*_) × *F*_*i*_. *U*_*i*_ is the CPU utilization rate of *P*_*i*_. The parameter *F*_*i*_ is the CPU frequency of *P*_*i*_. Remaining available memory resource of physical host *P*_*i*_ is as follows: AM_*i*_ = TM_*i*_ − UM_*i*_ − RM. TM_*i*_ is the total memory size of *P*_*i*_. UM_*i*_ is the used memory size of *P*_*i*_. RM is the reserved memory size of the system. *P*_*i*_ can be a member of solutions only if AF_*i*_ > *h* and AM_*i*_ > *m*.

**Figure 1:**
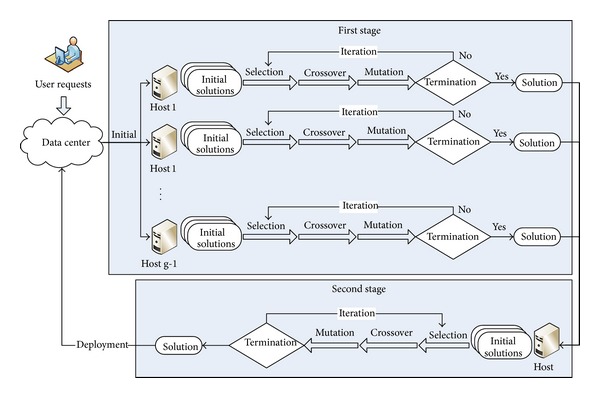
Distributed parallel genetic algorithm of VMs placement.

From the view of users, cloud center should select the physical hosts with more remaining resources to load the VMs with the objective of improving the QoS. From the view of cloud operators, cloud center should improve the utilization rates of resources and decrease the energy costs that aim at reducing the operating costs. Taken together, we assign the performance per watt to evaluation standard, namely, maximizing performance as well as minimizing energy costs. As shown in Figure 1, the idea of DPGA is divided into two stages. In the first stage, genetic algorithm is executed in parallel on *g* selected physical hosts. We select initial populations dispersedly and averagely by a certain step size in solution space for these physical hosts. Selection process chooses the solution vectors according to the probability which is proportional to the fitness value. Then the algorithm crosses the selected solution vectors and mutates the crossed solution vectors in the direction conducive to the fitness value. After crossover and mutation process, the algorithm iterates the first stage until it meets the iterative terminal conditions. In the second stage, the algorithm collects the solutions obtained from each selected physical host in the first stage, and then it executes the genetic algorithm again as in the first stage with collected solutions as initial population.

### 3.1. Initial Population Generation in the First Stage

Instead of random way, we assign the initial population for higher coverage rate in the initial population generating process. Initial vector set *w*/*n* as jump volume among the vector members. We select *g* initial vectors as initial solution vectors and set *w*/*n*/*g* as jump volume among the initial solutions. We set *w*/*n*/*g*/*g* as jump volume among the initial solutions of the selected physical hosts. To ensure the algorithm executing correctly, in this paper, we assume that *w*/*n*/*g*/*g* > 1. In physical host *x*  (0 ≤ *x* < *g*), the vector member *Q*_*xyz*_  (0 ≤ *z* < *n*) of initial solution vector *S*_*xy*_  (0 ≤ *y* < *g*) is as follows:

(1) $$\begin{array}{c} Q_{xyz}=\{\begin{array}{cc} P_{x\times w/n/g/g+y\times w/n/g+z\times w/n} & \\ x\times w/n/g/g+y\times w/n/g+z\times w/n\leq w, & \\ P_{x\times w/n/g/g+y\times w/n/g+z\times w/n-w} & \\ x\times w/n/g/g+y\times w/n/g+z\times w/n>w. & \end{array} \end{array}$$

Number *x* is the serial number of the physical host which is selected to execute the first stage algorithm. Number *y* is the solution vector serial number of the physical host *x*. Number *z* is the vector member serial number of the solution vector *y*.

For instance, we set *w* = 1000, *n* = 10, and *g* = 4. The initial population is showed in Table 1.

### 3.2. Fitness Calculation

We assign the performance per watt as fitness value. The performance increment of physical host *Q*_*xyz*_ is recorded as Δ*F*_*xyz*_ × *T*. Δ*F*_*xyz*_ is the CPU frequency increment of physical host *Q*_*xyz*_ and *T* is the VM work time. The energy consumption increment of physical host *Q*_*xyz*_ is recorded as Δ*E*_*xyz*_. The VCPU frequencies of placement VMs are (*h*_0_, *h*_1_, *h*_2_,…, *h*_*n*−1_) Hz, so Δ*F*_*xy*0_ = *h*_0_, Δ*F*_*xy*1_ = *h*_1_,…, Δ*F*_*xy*(*n*−1)_ = *h*_*n*−1_. The relationship among energy, voltage, and frequency in CMOS circuits [27] is related by

(2) $$\begin{array}{c} E=C\times F\times V^{2}\times T,\;\;F=K\times \frac{{\left(V-Vt\right)}^{2}}{V}, \end{array}$$

where *E* is energy consumption, *C* is CPU circuit switching capacity, *F* is CPU frequency, *V* is CPU voltage, *K* is a factor which depends on technology, and *Vt* is CPU threshold voltage. By formula (2), we can get the relationship between voltage and frequency as follows:

(3) $$\begin{array}{c} V=\sqrt{\frac{F\times Vt}{K}+\frac{F^{2}}{4\times K^{2}}}+Vt+\frac{F}{2\times K}. \end{array}$$

We can also get the energy consumption increment of physical host *Q*_*xyz*_ as follows:

(4) ΔExyz=Cxyz×(Fxyz+hz) ×((Fxyz+hz)×VtxyzKxyz+(Fxyz+hz)24×Kxyz2+Vtxyz+Fxyz+hz2×Kxyz)2×T −Cxyz×Fxyz×(Fxyz×VtxyzKxyz+Fxyz24×Kxyz2+Vtxyz+Fxyz2×Kxyz)2×T.

It updates the *F*_*xyz*_ = *F*_*xyz*_ + *h*_*z*_ dynamically and temporarily after Δ*E*_*xyz*_ calculation. The updated *F*_*xyz*_ only works in the process of the fitness value calculation for current solution vector. The fitness value of the algorithm is the ratio of the incremental performance and incremental power consumption after deploying the VMs according to the solution vector. Thus, the fitness value *I*_*xy*_ of the solution vector *S*_*xy*_ in the proposed VM placement strategy can be expressed as follows:

(5) Ixy=∑z=0n−1ΔFxyz×T∑z=0n−1ΔExyz=∑z=0n−1hz ×(∑z=0n−1Cxyz×(Fxyz+hz)   ×((Fxyz+hz)×VtxyzKxyz+(Fxyz+hz)24×Kxyz2      +Vtxyz+Fxyz+hz2×Kxyz)2    −Cxyz×Fxyz    ×(Fxyz×VtxyzKxyz+Fxyz24×Kxyz2      +Vtxyz+Fxyz2×Kxyz)2)−1.

### 3.3. Selection, Crossover, and Mutation in the First Stage

Selecting operations choose the solution vectors according to the probability in the direction proportional to the fitness value. The selected solution vectors with higher fitness value will get more opportunities to be inherited by succeeding generation. The selection probability *β*_*xy*_ of solution vector *S*_*xy*_ is as follows:

(6) $$\begin{array}{c} \beta_{xy}=\frac{I_{xy}}{\sum_{a=0}^{g-1}I_{xa}}. \end{array}$$

The selection probability area *α*_*xy*_ of solution vector *S*_*xy*_ between 0 and 1 is as follows:

(7) $$\begin{array}{c} \alpha_{xy}=\{\begin{array}{cc} \left[0,\beta_{xy}\right), & y=0,\\ \left[\overset{y-1}{\underset{a=0}{\sum }}\beta_{xa},\overset{y}{\underset{a=0}{\sum }}\beta_{xa}\right], & 0<y<g-1,\\ \left(\overset{y-1}{\underset{a=0}{\sum }}\beta_{xa},1\right], & y=g-1. \end{array} \end{array}$$

Then selection process generates *g* random numbers between 0 and 1. It selects *g* solution vectors according to *g* random numbers which appear in probability area.

In crossover process, we use the multipoint crossover method with self-adaptive crossover rate [28]. We set the initial crossover rate with 1. Firstly, crossover process calculates the crossover rate for the solution vectors of current generation. We record *ζ* ^pre^ as the crossover rate of previous generation solution vectors.

The average fitness value of current generation solution vectors is as follows:

(8) $$\begin{array}{c} I_{x\;\text{average}}=\frac{\sum_{i=0}^{g-1}I_{xi}}{g}. \end{array}$$

*I*_*xi*_ is the fitness value of the current generation solution vector. The average fitness value of previous generation solution vectors is as follows:

(9) $$\begin{array}{c} I_{x\;\text{average}}^{\text{pre}}=\frac{\sum_{i=0}^{g-1}I_{xi}^{\text{pre}}}{g}. \end{array}$$

*I*_*xi*_^pre^ is the fitness value of the previous generation solution vector. The crossover rate *ζ* of the current generation solution vectors is as follows:

(10) $$\begin{array}{c} \zeta =\zeta^{\text{pre}}\times \left(1-\frac{I_{x\;\text{average}}-I_{x\;\text{average}}^{\text{pre}}}{I_{x\;\text{average}}^{\text{pre}}}\right). \end{array}$$

We assume that *I*_*x*max⁡_ is the largest fitness value of the solution vectors. Crossover process uses the random mating strategy for mating the population. For each pair of mating solution vectors, we assume that *I*_*xy*_ is the bigger fitness value of the two solution vectors. The crossover rate *ζ*_*xy*_ of this pair of mating solution vectors is as follows:

(11) $$\begin{array}{c} \zeta_{xy}=\zeta \times \frac{I_{x\max}-I_{xy}}{I_{x\max}-I_{x\;\text{average}}}. \end{array}$$

If this pair of mating solution vectors needs to be crossed according to crossover rate *ζ*_*xy*_, crossover process generates *n* random numbers of 0 or 1. The position will be the crossover point if the random number is 1. The process crosses this pair of mating solution vectors according to the crossover points.

We use the multipoint mutation method with self-adaptive mutation rate [28] as in the crossover process. Firstly, the mutation process calculates the mutation rate for the solution vectors. We assume that *I*_*x*max⁡_ is the largest fitness value of the solution vectors. *I*_*xy*_ is the fitness value of solution vector *S*_*xy*_. The mutation rate *δ*_*xy*_ of *S*_*xy*_ is as follows:

(12) $$\begin{array}{c} \delta_{xy}=\frac{\zeta }{n}\times \frac{I_{x\max}-I_{xy}}{I_{x\max}-I_{x\;\text{average}}}. \end{array}$$

Then the mutation process sets the mutation points for *S*_*xy*_ according to mutation rate *δ*_*xy*_. If the mutation process sets the point as a mutation point, it records the related number with 1; otherwise it records the related number with 0. The solution vector is an incorrect solution after crossover if the number *θ* of a physical host that appears in solution vector is bigger than the number *φ* of VMs that can be loaded in this physical host. In this case, we set *θ*-*φ* mutation points (set the related number with 1) randomly on the position of the physical host in solution vector. For example, there are two solution vectors (*P*_5_, *P*_14_, *P*_54_, *P*_189_, *P*_201_, *P*_323_, *P*_405_, *P*_667_, *P*_701_, *P*_899_) and (*P*_88_, *P*_103_, *P*_166_, *P*_255_, *P*_323_, *P*_323_, *P*_391_, *P*_405_, *P*_653_, *P*_878_), the crossover point is 8, and then the solution vectors after crossover are (*P*_5_, *P*_14_, *P*_54_, *P*_189_, *P*_201_, *P*_323_, *P*_405_, *P*_405_, *P*_653_, *P*_878_) and (*P*_88_, *P*_103_, *P*_166_, *P*_255_, *P*_323_, *P*_323_, *P*_391_, *P*_667_, *P*_701_, *P*_899_). The solution vector (*P*_5_, *P*_14_, *P*_54_, *P*_189_, *P*_201_, *P*_323_, *P*_405_, *P*_405_, *P*_653_, *P*_878_) is an incorrect solution if the remaining available resources of *P*_405_ only can load one VM. So we set *P*_405_ as mutation point.

After determining the mutation points, the mutation process continues to mutate the mutation points. In initial population generation process, we take the coverage of the solution space into consideration, so the mutation process mutates the mutation point with the scope of *w*/*n*/*g*/*g*. As for *P*_*i*_, the mutation interval is as follows:

(13) [Pi−w/n/g/g+w,Pw−1]∪[P0,Pi+w/n/g/g]i−w/n/g/g<0,[Pi−w/n/g/g,Pi+w/n/g/g]i−w/n/g/g≥0, i+w/n/g/g≤w−1,[Pi−w/n/g/g,Pw−1]∪[P0,Pi+w/n/g/g−(w−1)]i+w/n/g/g>w−1.

Because of the indeterminacy of the mutation points, mutation process mutates the mutation points according to the sequence of the mutation points. According to the nonmutation points (the relevant position of random number is 0), mutation process updates the information of members in mutation interval and deletes the members of mutation interval if the remaining resources of relevant physical hosts cannot satisfy the requirement of users. The number of alternative mutation physical hosts after updating the mutation interval is *l*. The alternative mutation physical hosts are expressed as (*P*_0_′, *P*_1_′, *P*_2_′,…, *P*_*l*−1_′). If *l* = 0, the mutation process randomly selects a mutation physical host that can satisfy the requirements of users from solution space. If *l* > 0, the mutation process selects a physical host from mutation interval proportional to the benefit of the fitness value to mutate the mutation point.

If physical host *P*_*i*_′ loads the VM, the performance per watt *I*_*i*_′ is as follows:

(14) Ii′=(h) ×(Ci×(Fi+h) ×((Fi+h)×VtiKi+(Fi+h)24Ki2+Vti+Fi+h2Ki)2−Ci×Fi×(Fi×VtiKi+Fi24Ki2+Vti+Fi2Ki)2)−1.

The selection probability *β*_*i*_′ of alternative mutation physical host *P*_*i*_′ is as follows:

(15) $$\begin{array}{c} \beta_{i}^{\prime }=\frac{I_{i}^{\prime }}{\sum_{j=0}^{l-1}I_{j}^{\prime }}. \end{array}$$

The probability area *α*_*i*_′ of alternative mutation physical host *P*_*i*_′ between 0 and 1 is as follows:

(16) $$\begin{array}{c} \alpha_{i}^{\prime }=\{\begin{array}{cc} \left[0,\beta_{i}^{\prime }\right), & i=0,\\ \left[\overset{i-1}{\underset{a=0}{\sum }}\beta_{a}^{\prime },\overset{i}{\underset{a=0}{\sum }}\beta_{a}^{\prime }\right], & 0<i<l-1,\\ \left(\overset{i-1}{\underset{a=0}{\sum }}\beta_{a}^{\prime },1\right], & i=l-1. \end{array} \end{array}$$

Then mutation process generates a random number between 0 and 1. It selects an alternative physical host according to the probability area in which the random number appeared. After mutating the mutation point, mutation process sets the relevant position number of solution vector with 0.

### 3.4. Iteration and Termination in the First Stage

After the mutation process, the algorithm judges whether it reaches the iterative termination conditions of the first stage of DPGA. If so, the algorithm stops iteration in the first stage; otherwise it continues the iteration. The solution vector with the maximum fitness value is the optimal solution vector in the first stage. The iterative termination conditions of the first stage are as follows.

(1) Iterative times attain the preset maximum iterative times in the first stage. We set the maximum iterative times in the first stage with *τ*. The value of *τ* is related to *w* and *n*.
(2) The difference between the largest fitness value and the average fitness value is less than a certain ratio of the average fitness value. We set the difference ratio of the second termination condition of the first stage with *μ*. We record the largest fitness value of the solution vectors as *I*_*x*max⁡_. Thus, the first stage of the algorithm will terminate if it satisfies the following formula:
  (17) $$\begin{array}{c} I_{x\max}\leq \left(1+\mu \right)\times I_{x\;\text{average}}. \end{array}$$
(3) The optimized proportion of the average fitness values between two adjacent generation populations is less than the preset ratio. We set the optimized proportion of the third termination condition of the first stage with σ. Thus, the first stage of the algorithm will terminate if it satisfies the following formula:
  (18) $$\begin{array}{c} I_{x\;\text{average}}\leq \left(1+\sigma \right)\times I_{x\;\text{average}}^{\text{pre}}. \end{array}$$

### 3.5. Genetic Algorithm in the Second Stage

After completing the iteration in the first stage of DPGA, the algorithm collects the solution vectors obtained from the first stage as the initial population in the second stage. The selection process in the second stage chooses the solution vectors in the same way as in the first stage. We also use the multipoint crossover method with self-adaptive crossover rate as in the first stage.

The average fitness value of current generation solution vectors in the second stage is as follows:

(19) $$\begin{array}{c} I_{\text{average}}^{\prime }=\frac{\sum_{i=0}^{g-1}I_{i}^{\prime }}{g}. \end{array}$$

*I*_*i*_′ is the fitness value of the current generation solution vector in the second stage. The average fitness value of previous generation solution vectors in the second stage is as follows:

(20) $$\begin{array}{c} I_{\text{average}}^{{\text{pre}}^{\prime }}=\frac{\sum_{i=0}^{g-1}I_{i}^{{\text{pre}}^{\prime }}}{g}. \end{array}$$

*I*_*i*_^pre′^ is the fitness value of the previous generation solution vector in the second stage. The crossover rate *ζ*′ of the current generation solution vectors in the second stage is as follows:

(21) $$\begin{array}{c} \zeta^{\prime }=\zeta^{{\text{pre}}^{\prime }}\times \left(1-\frac{I_{\text{average}}^{\prime }-I_{\text{average}}^{{\text{pre}}^{\prime }}}{I_{\text{average}}^{{\text{pre}}^{\prime }}}\right). \end{array}$$

*ζ* ^pre′^ is the crossover rate of the previous generation solution vectors in the second stage. *I*_*i*_′ is the fitness value of the current generation solution vector in the second stage. *I*_*i*_^pre′^ is the fitness value of the previous generation solution vector in the second stage. The crossover rate *ζ*_*x*_′ of the pair of the mating solution vectors in the second stage is as follows:

(22) $$\begin{array}{c} \zeta_{x}^{\prime }=\zeta^{\prime }\times \frac{I_{\max}^{\prime }-I_{x}^{\prime }}{I_{\max}^{\prime }-I_{\text{average}}^{\prime }}. \end{array}$$

*I*_max⁡_′ is the largest fitness value of the solution vectors in the second stage. *I*_*x*_′ is the bigger fitness value of the pair of the mating solution vectors in the second stage.

The mutation process uses the multipoint mutation method with self-adaptive mutation rate and confirms the mutation points as the way it used in the first stage. The mutation rate *δ*_*x*_′ of *S*_*x*_ in the second stage is as follows:

(23) $$\begin{array}{c} \delta_{x}^{\prime }=\frac{\zeta^{\prime }}{n}\times \frac{I_{\max}^{\prime }-I_{x}^{\prime }}{I_{\max}^{\prime }-I_{\text{average}}^{\prime }}. \end{array}$$

*I*_max⁡_′ is the largest fitness value of the solution vectors in the second stage. *I*_*x*_′ is the fitness value of solution vector *S*_*x*_′ in the second stage. After confirming the mutation points in the second stage, being different from the process in the first stage, it mutates the mutation points with the scope of the whole solution space. Because of the indeterminacy of the mutation points, mutation process mutates the mutation points according to the sequence of the mutation points. Firstly, according to the nonmutation points, mutation process updates the information of all solution space members and deletes the members of mutation interval if the remaining resources of relevant physical hosts cannot satisfy the requirement of users. Then the mutation process mutates the mutation points in the same way as in the first stage.

After the mutation process in the second stage, the algorithm judges whether it reaches the iterative termination conditions of the second stage of DPGA. If so, the algorithm stops iteration in the second stage; otherwise it continues the iteration. The solution vector with the maximum fitness value is the optimal solution vector. The iterative termination conditions of the second stage are as follows.

(1) Iterative times attain the preset maximum iterative times in the second stage. Because the solution vectors obtained from the first stage are relative optimal solutions, we decrease the maximum iterative times accordingly in the second stage. We set the maximum iterative times in the second stage with *τ*/*g*.
(2) The difference between the largest fitness value and the average fitness value is less than a certain ratio of the average fitness value. As the result of the fact that the solution vectors obtained from the first stage are relative optimal solutions, we decrease the difference ratio accordingly in the second stage. We set the different ratio of the second termination condition of the second stage with *μ*/*g*. We record the largest fitness value of the solution vectors as *I*_max⁡_′. Thus, the second stage of the algorithm will terminate if it satisfies the following formula:
  (24) $$\begin{array}{c} I_{\max}^{\prime }\leq \left(1+\mu /g\right)\times I_{\text{average}}^{\prime }. \end{array}$$
(3) The optimized proportion of the average fitness values between two adjacent generation populations is less than the preset ratio. We decrease the optimized proportion accordingly in the second stage in consequence of the fact that the solution vectors obtained from the first stage are relative optimal solutions. We set the optimized proportion of the third termination condition of the second stage with *σ*/*g*. The second stage of the algorithm will terminate if it satisfies the following formula:
  (25) $$\begin{array}{c} I_{\text{average}}^{\prime }\leq \left(1+\sigma /g\right)\times I_{\text{average}}^{{\text{pre}}^{\prime }}. \end{array}$$

## 4. Evaluation

In order to simulate a dynamic cloud platform, we utilize a cloud simulator named CloudSim toolkit [29], version 3.0.3. The CloudSim framework can create different kinds of entities and remove data center entities at run time. The CloudSim framework can also calculate the status information of entities such as resource utilization and power consumption during the simulation period. We choose 6 kinds of host models as shown in Table 2 for CloudSim platform in the experiments.

According to Table 3, we need to create power model classes for each kind of host models to calculate the power consumption of the hosts in CloudSim platform [30].

In the experiments, DPGA needs some parameters of the hosts. The CloudSim platform does not provide the parameters *C*, *K*, and *Vt* of the hosts which should have been obtained from the hardware providers. Therefore we need to calculate the approximate values of the parameters. Firstly, we pick up two groups of core voltage and core frequency for each kind of host model, and then we calculate their power consumption by the CloudSim platform. Finally, we utilize the matlab [31] to solve the multiple equations established by formula (2) according to the information of Table 4. The values of parameters are showed in Table 4.

The class PowerHost of the CloudSim platform does not contain the member variables of *C*, *K*, and *Vt*. We create a new class which extends the class PowerHost by adding the member variables of *C*, *K*, and *Vt* so that the entities in the experiments can record the information of parameters for DPGA. In the experiments, a data center consisting of *w* hosts is created. These hosts are averagely composed of the above 6 kinds of host models. Then the data center creates *d* VMs according to Table 5 averagely with the full utilization model as the original loads of the data center.

In the experiments, the data center creates *n* VMs according to Table 6 averagely as the requirements of users with full utilization model.

### 4.1. Performance per Watt with Different Original Loads

**Figure 2:**
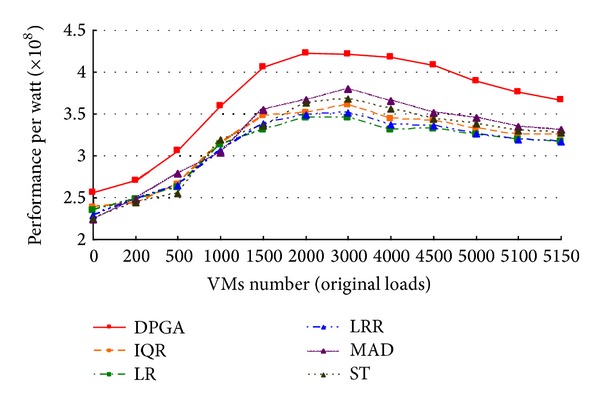
Comparison of performance per watt with different original loads.

In this experiment, we set the hosts number of the data center *w* = 1600 and the VMs number *n* = 10 as the requirements of users. We adjust the VMs number *d* as the original loads from 0 to 5000 and allocate these VMs to the hosts randomly. It represents different load levels of the data center. All idle hosts are switched to Sleep state. The experiment is designed for verifying the efficiency of DPGA in performance per watt of a cloud center under different original loads. In this scenario, we compare performance per watt of DPGA with ST (static threshold) which sets the utilization threshold to 0.9, IQR (interquartile range) which sets the safety parameter to 1.5, LR (local regression) which sets the safety parameter to 1.2, LRR (local regression robust) which sets the safety parameter to 1.2, and MAD (median absolute deviation) which sets the safety parameter to 2.5 [30]. As illustrated in Figure 2, DPGA placement strategy for VMs deployment under different original loads gets higher performance per watt than other placement strategies. Further, when *d* ≤ 1000, namely, the data center under an approximate idle state, performance per watt of DPGA placement strategy increases rapidly. When 1000 < *d* ≤ 2000, namely, the data center under a low loading state, performance per watt of DPGA placement strategy increases at a relatively flat rate. When 2000 < *d* ≤ 4000, namely, the data center under a moderate loading state, performance per watt of DPGA placement strategy is relatively stable. When *d* > 4000, namely, the data center under an overloading state, performance per watt of DPGA placement strategy begins to decline gradually. This is because the hosts under the state from idle to load or under the overload states consume more power than the hosts under the state of a certain load. In conclusion, DPGA has a better performance per watt and is relatively more stable because DPGA placement strategy is the heuristic approach. It takes the performance per watt as evaluation standard and tends towards stability by two step iterations.

### 4.2. Performance per Watt with Different User Requests

**Figure 3:**
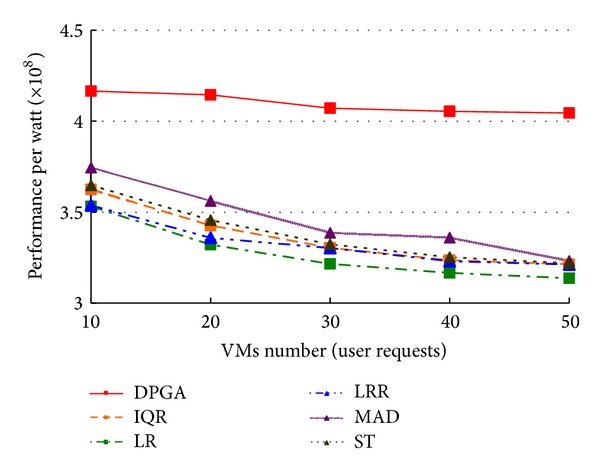
Comparison of performance per watt with different user requests.

In this experiment, we set the hosts number of the data center *w* = 1600 and the VMs number *d* = 3000 as the original loads. Then we allocate these VMs to the hosts randomly. We adjust the VMs number *n* as the requirements of users from 10 to 50. All idle hosts are switched to Sleep state. The experiment is designed for verifying the efficiency of DPGA in performance per watt of a cloud center with different requirements of users. In this scenario, we compare performance per watt of DPGA with ST, IQR, LR, LRR, and MAD that take the same parameters as the experiment in Section 4.1. As illustrated in Figure 3, DPGA placement strategy for VMs deployment with different requirements of users gets higher performance per watt than other placement strategies. Further, with the increase of the requirements of users, DPGA placement strategy for VMs deployment gets more stable performance per watt than other placement strategies.

### 4.3. Performance per Watt with Different State of Idle Hosts

**Figure 4:**
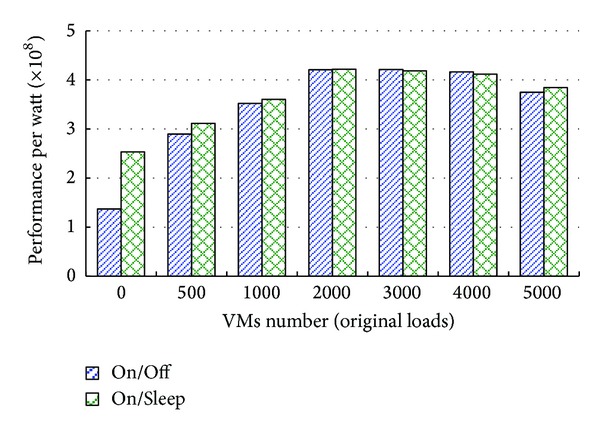
Comparison of performance per watt with different state of idle hosts.

In this experiment, we set the hosts number of the data center *w* = 1600 and the VMs number *n* = 10 as the requirements of users. We adjust the VMs number *d* as the original loads from 0 to 5000 and allocate these VMs to the hosts randomly. It represents different load levels of the data center. There are two policies to be formulated for idle hosts. The first policy is On/Off policy, wherein all idle hosts are switched off. The second policy is On/Sleep policy, wherein all idle hosts are switched to Sleep state. The experiment is designed for verifying the efficiency of DPGA in performance per watt of a cloud center with different policies for idle hosts. In this scenario, we compare performance per watt of DPGA with On/Off policy for idle hosts and DPGA with On/Sleep policy for idle hosts. As illustrated in Figure 4, DPGA placement strategy for VMs deployment with On/Sleep policy gets higher performance per watt than DPGA placement strategy with On/Off policy when the data center is under an approximate idle state. DPGA placement strategy for VMs deployment with On/Sleep policy gets approximately the same performance per watt as DPGA placement strategy with On/Off policy when the data center is under a loading state. This is because the idle hosts at Sleep state consume certain power while the turned-off idle hosts do not consume any power. Therefore DPGA placement strategy for VMs deployment is more suitable for the cloud center under a loading state.

### 4.4. Actual and Theoretical Values of Performance per Watt

**Figure 5:**
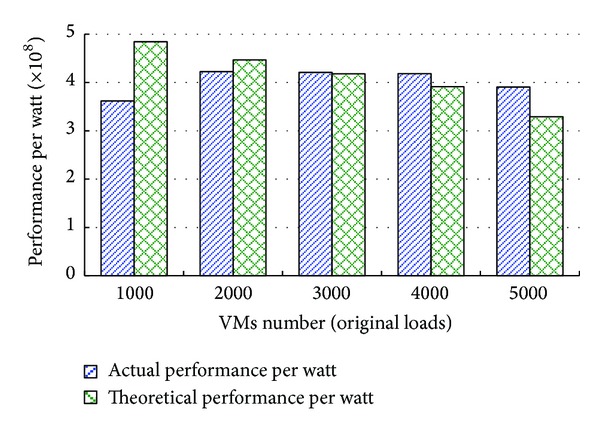
Comparison of actual performance per watt and theoretical performance per watt.

In this experiment, we set the hosts number of the data center *w* = 1600 and the VMs number *n* = 10 as the requirements of users. We adjust the VMs number *d* as the original loads from 500 to 5000 and allocate these VMs to the hosts randomly. It represents different load levels of the data center. All idle hosts are switched to Sleep state. In this scenario, we compare actual performance per watt of DPGA with theoretical performance per watt of DPGA calculated by formula (5). As illustrated in Figure 5, theoretical performance per watt is higher than actual performance per watt when the data center is under a low loading state. Theoretical performance per watt is approximately the same as actual performance per watt when the data center is under a moderate loading state. Theoretical performance per watt is lower than actual performance per watt when the data center is under an overloading state. This is because the hosts under a moderate loading state can calculate a relatively more accurate value of power consumption by DVFS formula than the hosts under a low loading state or an overloading state. In conclusion, DPGA placement strategy for VMs deployment is more suitable for the cloud center under a moderate loading state.

## 5. Conclusion

In this paper, we present the design, implementation, and evaluation of a distributed parallel genetic algorithm of virtual machine placement strategy on cloud platform. The algorithm is divided into two stages to get a better and more accurate solution. We assign the performance per watt as evaluation standard. We use the multipoint crossover method with self-adaptive crossover rate and the multipoint mutation method with self-adaptive mutation rate in the proposed approach. DPGA executes the first stage genetic algorithm with selected initial subpopulation and puts the solutions obtained into the second stage genetic algorithm as initial population. Then it finally gets a relatively optimal solution from the second stage. The experimental results show that our approach is an efficient, stable, and effective placement strategy for VM deployment.

To further improve the performance of placement strategy for VM deployment, there are also many problems that need to be solved in the future. The number of parallel executions in the first stage *g* should be related to the size of solution space *w* and the number of deployment VMs *n*. We plan to assign the value of *g* according to *w* and *n*. In population initialization process, we select initial subpopulation from solution space dispersedly and averagely. In crossover and mutation process, we use the multipoint crossover method with self-adaptive crossover rate and the multipoint mutation method with self-adaptive mutation rate. We plan to optimize the algorithm in detail. In the judgment of iterative termination conditions, the maximum iteration times should be related to the size of solution space *w* and the number of deployment VMs *n*. We plan to assign the maximum iteration times according to *w* and *n*. There are also two open questions on the termination of the two stages. One is to determine the difference ratio between the largest fitness value and average fitness value, and the other one is to determine the optimized proportion of the average fitness values between two adjacent generation populations. In this paper, to ensure that the algorithm is executed correctly, we assume that *w*/*n*/*g*/*g* > 1. In order to execute the algorithm efficiently in the case of *w*/*n*/*g*/*g* ≤ 1, we plan to combine our approach with other methods. Our approach is appropriate for the case that all physical hosts of solution space are in a fast LAN and in the same network environment. We plan to extend our approach to WAN and different network environment. Our approach uses the parallel genetic algorithm. We plan to use other heuristic algorithms such as ant colony algorithm, bee colony algorithm, and particle swarm optimization to implement placement strategy of VMs deployment and compare their performance.

## Figures and Tables

**Table 1:** An example of initial population in the first stage.

Physical host (*x*)	Initial solution vector (*y*)	Member of initial solution vector
0	0	(*P*_0_, *P*_100_, *P*_200_, *P*_300_, *P*_400_, *P*_500_, *P*_600_, *P*_700_, *P*_800_, *P*_900_)
	1	(*P*_25_, *P*_125_, *P*_225_, *P*_325_, *P*_425_, *P*_525_, *P*_625_, *P*_725_, *P*_825_, *P*_925_)
	2	(*P*_50_, *P*_150_, *P*_250_, *P*_350_, *P*_450_, *P*_550_, *P*_650_, *P*_750_, *P*_850_, *P*_950_)
	3	(*P*_75_, *P*_175_, *P*_275_, *P*_375_, *P*_475_, *P*_575_, *P*_675_, *P*_775_, *P*_875_, *P*_975_)

1	0	(*P*_6_, *P*_106_, *P*_206_, *P*_306_, *P*_406_, *P*_506_, *P*_606_, *P*_706_, *P*_806_, *P*_906_)
	1	(*P*_31_, *P*_131_, *P*_231_, *P*_331_, *P*_431_, *P*_531_, *P*_631_, *P*_731_, *P*_831_, *P*_931_)
	2	(*P*_56_, *P*_156_, *P*_256_, *P*_356_, *P*_456_, *P*_556_, *P*_656_, *P*_756_, *P*_856_, *P*_956_)
	3	(*P*_81_, *P*_181_, *P*_281_, *P*_381_, *P*_481_, *P*_581_, *P*_681_, *P*_781_, *P*_881_, *P*_981_)

2	0	(*P*_12_, *P*_112_, *P*_212_, *P*_312_, *P*_412_, *P*_512_, *P*_612_, *P*_712_, *P*_812_, *P*_912_)
	1	(*P*_37_, *P*_137_, *P*_237_, *P*_337_, *P*_437_, *P*_537_, *P*_637_, *P*_737_, *P*_837_, *P*_937_)
	2	(*P*_62_, *P*_162_, *P*_262_, *P*_362_, *P*_462_, *P*_562_, *P*_662_, *P*_762_, *P*_862_, *P*_962_)
	3	(*P*_87_, *P*_187_, *P*_287_, *P*_387_, *P*_487_, *P*_587_, *P*_687_, *P*_787_, *P*_887_, *P*_987_)

3	0	(*P*_18_, *P*_118_, *P*_218_, *P*_318_, *P*_418_, *P*_518_, *P*_618_, *P*_718_, *P*_818_, *P*_918_)
	1	(*P*_43_, *P*_143_, *P*_243_, *P*_343_, *P*_443_, *P*_543_, *P*_643_, *P*_743_, *P*_843_, *P*_943_)
	2	(*P*_68_, *P*_168_, *P*_268_, *P*_368_, *P*_468_, *P*_568_, *P*_668_, *P*_768_, *P*_868_, *P*_968_)
	3	(*P*_93_, *P*_193_, *P*_293_, *P*_393_, *P*_493_, *P*_593_, *P*_693_, *P*_793_, *P*_893_, *P*_993_)

**Table 2:** Host models for CloudSim platform in the experiments.

Host	CPU	Memory (G)
IBM System X3650 M4	2 × [Intel Xeon E5-2660 2200 MHz, 10 cores]	64
IBM System X3300 M4	2 × [Intel Xeon E5-2470 2300 MHz, 8 cores]	24
Dell PowerEdge R710	2 × [Intel Xeon X5675 3066 MHz, 6 cores]	24
Dell PowerEdge R610	2 × [Intel Xeon X5670 2933 MHz, 6 cores]	12
Acer Altos AR580 F2	4 × [Intel Xeon X4607 2200 MHz, 6 cores]	64
Acer Altos R380 F2	2 × [Intel Xeon X2650 2000 MHz, 8 cores]	24

**Table 3:** Benchmark results summary of host models.

Host	Power consumption for the different target loads (W)										
	0%	10%	20%	30%	40%	50%	60%	70%	80%	90%	100%
IBM System X3650 M4	52.7	80.5	90.3	100	110	120	131	143	161	178	203
IBM System X3300 M4	50.8	74.3	84.1	94.5	106	122	141	164	188	220	260
Dell PowerEdge R710	62.2	104	117	127	137	147	157	170	187	205	227
Dell PowerEdge R610	61.9	102	115	126	137	149	160	176	195	218	242
Acer Altos AR580 F2	109	155	170	184	197	211	226	252	280	324	368
Acer Altos R380 F2	52.9	77.1	85.4	94	102	110	124	141	162	186	215

**Table 4:** Parameters summary of host models.

Host	Core voltage (V)	Frequency (Hz)	Power (W)	Δ*E* (W)	*C* (F)	*K*	*Vt.* (V)
IBM System X3650 M4	0.806	800 ∗ 10^6^	106.364	85.272	0.501 ∗ 10^−12^	87.565 ∗ 10^9^	0.422
	1.172	2100 ∗ 10^6^	191.636				

IBM System X3300 M4	0.986	821.5 ∗ 10^6^	101.075	130.386	0.631 ∗ 10^−12^	57.787 ∗ 10^9^	0.512
	1.433	2135.9 ∗ 10^6^	231.461				

Dell PowerEdge R710	0.857	1066.4 ∗ 10^6^	131.781	85.647	0.526 ∗ 10^−12^	72.411 ∗ 10^9^	0.468
	1.246	2932.6 ∗ 10^6^	217.428				

Dell PowerEdge R610	0.906	980 ∗ 10^6^	129.754	96.781	0.566 ∗ 10^−12^	65.298 ∗ 10^9^	0.502
	1.317	2744 ∗ 10^6^	226.535				

Acer Altos AR580 F2	0.994	800 ∗ 10^6^	192.273	191.727	0.617 ∗ 10^−12^	85.299 ∗ 10^9^	0.521
	1.445	2100 ∗ 10^6^	348				

Acer Altos R380 F2	0.953	700 ∗ 10^6^	98	102.5	0.59 ∗ 10^−12^	55.441 ∗ 10^9^	0.514
	1.386	1900 ∗ 10^6^	200.5				

**Table 5:** VM models for loads of data center.

Item	VM models							
	Model 1	Model 2	Model 3	Model 4	Model 5	Model 6	Model 7	Model 8
VCPU (MHz)	1000 ∗ 1	1200 ∗ 2	1300 ∗ 2	1400 ∗ 4	1500 ∗ 4	1600 ∗ 6	1800 ∗ 6	2000 ∗ 8
RAM (M)	512	1024	1024	2048	2048	4096	4096	8192

**Table 6:** VM models for user requests.

Item	User request models									
	Model 1	Model 2	Model 3	Model 4	Model 5	Model 6	Model 7	Model 8	Model 9	Model 10
VCPU (MHz)	1000 ∗ 1	1100 ∗ 2	1300 ∗ 2	1400 ∗ 4	1500 ∗ 4	1600 ∗ 6	1700 ∗ 6	1800 ∗ 8	1900 ∗ 8	2000 ∗ 10
RAM (M)	256	512	512	1024	1024	2048	2048	4096	4096	8192
